# Targeting the Post-Irradiation Tumor Microenvironment in Glioblastoma via Inhibition of CXCL12

**DOI:** 10.3390/cancers11030272

**Published:** 2019-02-26

**Authors:** Frank A. Giordano, Barbara Link, Martin Glas, Ulrich Herrlinger, Frederik Wenz, Viktor Umansky, J. Martin Brown, Carsten Herskind

**Affiliations:** 1 Department of Radiation Oncology, Universitätsmedizin Mannheim, Medical Faculty Mannheim, Heidelberg University, 68167 Mannheim, Germany; barbara.link@medma.uni-heidelberg.de (B.L.); carsten.herskind@medma.uni-heidelberg.de (C.H.); 2 Division of Clinical Neurooncology, Department of Neurology and West German Cancer Center (WTZ), University Hospital Essen and German Cancer Consortium, Partner Site University Hospital Essen, University Duisburg-Essen, 45147 Essen, Germany; Martin.Glas@UK-Essen.de; 3 Division of Clinical Neurooncology, Department of Neurology, University of Bonn Medical Center, 53105 Bonn, Germany; Ulrich.Herrlinger@ukbonn.de; 4 CEO, University Medical Center Freiburg, 79110 Freiburg, Germany; frederik.wenz@uniklinik-freiburg.de; 5 Skin Cancer Unit, German Cancer Research Center (DKFZ), 69120 Heidelberg, Germany; v.umansky@dkfz.de; 6 Department of Dermatology, Venereology and Allergology, University Medical Center Mannheim, Ruprecht-Karl University of Heidelberg, 68167 Mannheim, Germany; 7 Department of Neurology, Stanford University School of Medicine, Stanford, CA 94305, USA; mbrown@stanford.edu

**Keywords:** glioblastoma, radiotherapy, CXCL12

## Abstract

Radiotherapy is a mainstay in glioblastoma therapy as it not only directly targets tumor cells but also depletes the tumor microvasculature. The resulting intra-tumoral hypoxia initiates a chain of events that ultimately leads to re-vascularization, immunosuppression and, ultimately, tumor-regrowth. The key component of this cascade is overexpression of the CXC-motive chemokine ligand 12 (CXCL12), formerly known as stromal-cell derived factor 1 (SDF-1). We here review the role of CXCL12 in recruitment of pro-vasculogenic and immunosuppressive cells and give an overview on future and current drugs that target this axis.

## 1. Introduction

Glioblastoma (GB, WHO Grade IV astrocytoma) is the most common malignant and most aggressive primary brain tumor. The incidence generally increases with age, and the median age of diagnosis is 64 years [1]. The age-adjusted incidence in the U.S. is approximately 3 per 100,000 persons, and survival time of patients diagnosed with GB is usually between 12 and 24 months, with less than 5% living up to 5 years [2]. Focal neurological deficits, symptoms of increased intracranial pressure, epilepsy, and cognitive dysfunction are prominent symptoms which may arise in any stage of the disease [3,4]. Key prognostic factors for survival include general factors such as age, clinical performance status and the extent of resection. 

The presence or absence of a promoter methylation of the O^6^-methylguanine DNA methyltransferase (MGMT) has been shown to be of specific relevance for outcome as it may predict the response to chemotherapy and overall prognosis as a confirmed prognostic biomarker [5,6]. Treatment of GBM requires a multidisciplinary approach and, for more than a decade, patients with adequate performance scores and tumors amenable to resection undergo surgery and then combined external-beam radiotherapy (EBRT) and chemotherapy with the alkylating agent temozolomide followed by maintenance temozolomide, the ‘Stupp protocol’ [7]. Elderly or frail patients may, depending on molecular markers (MGMT), alternatively receive either radiotherapy alone, temozolomide alone, or short-course radiotherapy with or without temozolomide [8,9,10]. 

A recent randomized clinical phase III trial has shown survival benefits after treatment with a portable, non-invasive device that delivers low-intensity, intermediate-frequency, alternating electric fields to the brain and acts by reversing tumor growth by inhibiting cell division [11]. This therapy, commonly referred to as Tumor-Treating Fields (TTF), has evolved as an additional treatment modality on top of maintenance temozolomide chemotherapy, which is usually initiated after radiochemotherapy. 

Despite their effectiveness in GB, all modalities exhibit characteristic adverse effects. Common complications from surgical resection are focal neurological deficits; radiotherapy induces vascular injury, radiation necrosis and gliosis and in patients with longer survival there is also a risk of long-term neurocognitive impairment [12]. Common adverse reactions to temozolomide are mostly limited to the many features of myelotoxicity (anemia, leukopenia, thrombocytopenia) but may also include more unspecific side effects such as nausea, skin rashes and liver toxicity [13,14,15,16]. 

The benefit of the current standard of care, surgical resection followed by radiotherapy and adjuvant chemotherapy, is modest. The Stupp regimen demonstrated a median survival of 14.6 months for treatment with radiotherapy plus temozolomide vs. 12.1 months with radiotherapy alone [7], which is still higher than an expected survival of approximately 7 months with best supportive care only [17]. Although the success of other ‘classical’ chemotherapies has been limited, the German CeTeG trial demonstrated that the addition of lomustine (CCNU) is beneficial in terms of overall survival in MGMT methylated patients [18]. Other approaches addressing classical cellular signaling pathways (epidermal growth factor receptor: EGFR; fibroblast growth factor receptor: FGFR; tyrosine-protein kinase c-Met: MET; platelet-derived growth factor receptor: PDGFR; phosphoinositide-3-kinase/protein kinase B/mammalian target of rapamycin: PI3K/AKT/mTOR; mitogen-activated protein kinase: MAPK) have failed for various reasons such as weak penetration of the blood brain barrier [19] or bypasses (e.g., resistance to EGFR therapy via insulin-like growth factor receptor (IGFR)-I signaling) and downregulation of pathways [20,21].

Almost all GB recur (mostly local) after first line treatment and, to date, no standard of care has been established for treating recurrent GB. Commonly applied treatment options are re-surgery (if applicable), re-irradiation, chemotherapy with CCNU or therapy with the angiogenesis inhibitor bevacizumab [22]. Almost all salvage options may be considered as palliative: (i) surgery may not tackle the complete extent of the (mostly dispersed) tumor, (ii) EBRT cannot be applied in the same intensity as in the first line due to the limited tolerance of brain tissue towards radiation and (iii) chemotherapy with temozolomide or CCNU is rendered ineffective by the repair enzyme MGMT. 

## 2. Tumor Environment and Immunosuppression in the Brain

It has been controversially discussed for decades if and how the central nervous system (CNS) could be a subject of active immunosurveillance and vigorous immune responses [23]. However, the recent finding that T cells primed by antigen presenting cells in cervical lymph nodes could reach the brain via connecting lymphatic vessels [24] suggests that although the CNS is clearly an immunologically distinct site, its immune microenvironment offers opportunities to implement immunotherapy for brain tumors [25]. Nevertheless, GB is considered as a poorly immunogenic [26], “cold” tumor with only few tumor-infiltrating lymphocytes (TILs) that frequently express markers of exhaustion [27,28]. Furthermore, GB could suppress TIL functions through increased concentrations of extracellular potassium typical for hypoxic tumor areas [29]. In contrast, brain tumors were found to be infiltrated by large numbers of myeloid cells such as microglia, macrophages, and myeloid-derived suppressor cells (MDSCs) [30].

**Figure 1:**
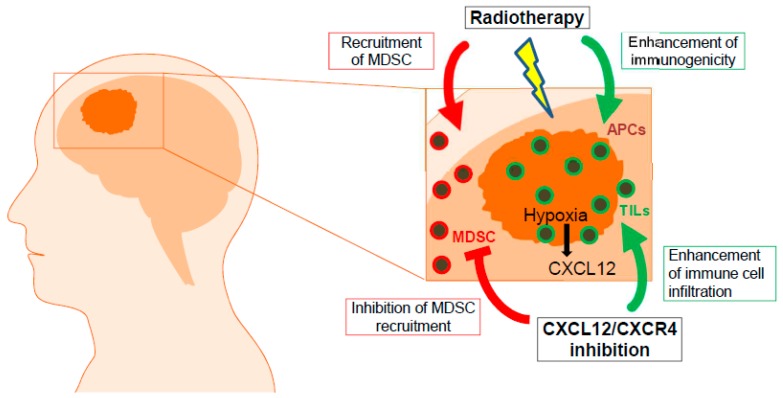
Components of the glioblastoma tumor microenvironment. APC: antigen-presenting cells; MDSC: myeloid-derived suppressor cells; TIL: tumor-infiltrating lymphocytes; CXCL12: C-X-C chemokine ligand 12; CXCR4: C-X-C chemokine receptor 4.

It has been demonstrated that MDSCs play a critical role in the development of an immunosuppressive tumor microenvironment [31,32,33] (Figure 1). This extremely heterogeneous population of myeloid cells was found to efficiently inhibit T-cell mediated anti-tumor reactivity through various mechanisms [31,32,34]. In mice, MDSCs express Gr1 and CD11b surface molecules and consist of two major subsets: polymorphonuclear CD11b^+^Ly6G^+^Ly6C^lo/−^ (PMN-MDSCs) and monocytic CD11b^+^Ly6G^−^Ly6C^hi^ (M-MDSCs) [31,32,35]. In humans, M-MDSCs are defined as CD11b^+^CD14^+^CD15^−^HLA-DR^low/−^ cells. Human PMN-MDSCs are characterized as CD11b^+^CD14^−^CD15^+^HLA^−^DR^−^ or CD11b^+^CD14^−^CD66b^+^ [36,37]. In addition, a subset of more immature human MDSCs characterized as Lin^−^ (including CD3, CD14, CD15, CD19, CD56) HLA-DR-CD33^+^ cells were defined as early-stage MDSCs (eMDSCs) [35]. Recently, a lectin-type oxidized LDL receptor-1 (LOX-1) has been proposed as a new marker for human PMN-MDSCs to distinguish them from neutrophils [38].

MDSCs derive from bone marrow hematopoietic precursors due to the altering of myelopoiesis induced by chronic inflammatory mediators such as granulocyte-macrophage colony-stimulating factor (GM-CSF), granulocyte colony-stimulating factor (G-CSF), macrophage colony-stimulating factor (M-CSF), stem cell factor (SCF), Vascular Endothelial Growth Factor (VEGF), Interleukin (IL) 6 and 1β [31,32,37,39]. The signaling mainly involves the signal transducer and activator of transcription 3 (STAT3), preventing MDSC differentiation and promoting their proliferation [40,41]. However, the induction of MDSC activity in tumor lesions is provided by pro-inflammatory molecules such as interferon (IFN)-γ, IL-1β, IL-4, Tumor necrosis factor (TNF)-α, toll-like receptor (TLR) ligands, prostaglandin (PGE) E2 and is mediated by STAT1, STAT6 and nuclear factor (NF)-κB transcription factors [31,40,42].

The migration of MDSCs into the tumor microenvironment is mediated by chemokines produced by tumor and host cells. It has been reported that the trafficking of M-MDSCs occurred via an interaction between chemokine (C-C motif) ligand CCL2 and its receptors CCR2, CCR4, and CCR5 [43]. Moreover, the targeting of CCL2/CCR2 axis with antibodies carlumab showed a modest activity as a single-agent therapeutic in patients with metastatic, castration-resistant prostate cancer [44]. On the other hand, the enhanced production of C-X-C motif receptor (CXCR) 2 ligands supported the migration of PMN-MDSCs to the tumor site [45]. Previous publications have indicated that CCL5 supported the tumor growth, invasion, and angiogenesis, as well as immune cell recruitment to the tumor microenvironment via the interaction with CCR5 [46].

In patients with glioblastoma, the frequency of circulating M-MDSCs and PMN-MDSCs was increased as compared to healthy donors [47]. In addition, most of tumor infiltrating MDSC represented PMN-MDSC characterized as CD15^+^HLA^−^DR^−^ cells [47]. Another study revealed that that glioblastoma patients displayed higher frequencies of circulating MDSC than age-matched healthy donors and patients with other tumor entities [48]. Interestingly, the majority of these cells were CD15^+^ CD14^−^ PMN-MDSCs. Analyzing a cohort of 52 glioblastoma patients, it was reported a strong accumulation of both PMN- and M-MDSC as compared healthy donors [49]. Importantly, reduced MDSC frequency in the peripheral blood of newly diagnosed glioblastoma was found to be associated with their extended survival, providing a basis for developing strategies to target MDSC in glioblastoma [50].

## 3. The Glioblastoma Microenvironment Switches from “Angiogenesis” to “Vasculogenesis”

A hallmark of glioblastoma is an abnormal and dysfunctional vasculature which in combination with the glycolytic environment leads to hypoxia [51]. It is now considered that severely hypoxic zones contribute to invasion by causing so called ‘pseudopalisades’ of cells to migrate away from the central hypoxia [52,53]. Radiotherapy (RT) is one of the key treatment modalities of glioblastoma and has been reported to cause blood vessel damage that may lead to additional tumor cell kill with sufficiently high radiation doses per fraction [54]. Although radiation-induced loss of blood vessels is widely considered a positive effect of RT, it initiates a chain of events that counteract tumor cell killing [55]. Depletion of microvasculature results in weakly perfused or non-perfused areas in which hypoxia-surviving cells respond with overexpression of the otherwise constitutively but only moderately expressed transcription factor hypoxia induced factor 1 alpha (HIF-1α). HIF-1α then induces expression of a wide variety of genes such as VEGF and angiopoietin 2 (ANGPT2), which in turn counteract hypoxia by promoting angiogenesis [56,57]. Besides these rather “directly” acting factors, HIF-1α also induces overexpression of CXC-motive chemokine ligand 12 (CXCL12), formerly known as stromal-cell derived factor 1 (SDF-1). CXCL12 in turn attracts hematopoietic progenitor cells that carry the chemokine (C-X-C motif) receptor 4 (CXCR4; also known as CD184 or fusin). 

## 4. The CXCL12/CXCR4/ACKR3 Axis in Inflammation and Vasculogenesis

Overexpression of CXCL12 and CXCR4 is associated with higher grade and poor prognosis of GBM [58,59]. A potentially important aspect of targeting CXCL12 is its ability to form a heterodimer with high mobility group B1 (HMGB1), a non-histone nuclear protein which acts as a damage associated molecular pattern (DAMP) molecule [60]. Thus, HMGB1 and other DAMP molecules (calreticulin, ATP, dsDNA) are released by damaged cells after irradiation with doses high enough to initiate an inflammatory response [61]. Heterodimerization of CXCL12 with HMGB1 is necessary for attracting monocytes to injured tissue, and migration of mouse embryo fibroblasts and leukocytes towards HMGB1 could be blocked by the CXCR4 inhibitor plerixafor (formerly known as AMD 3100) [62,63]. The redox state of HMGB1 determines which signal pathways are activated; thus, the reduced all-thiol form binds to CXCL12 in a heterocomplex which is more efficient in attracting lymphocytes than CXCL12 alone [60,64]. In relation to tumors, CXCR4 is present on a number of immune suppressor cells, including regulatory T cells (Treg) and myeloid-derived suppressor cells (MDSCs) [65].

The production of CXCL12 can be induced by prostaglandin E2 resulting in a strong enrichment of MDSCs in ovarian and gastric cancer microenvironment [66]. Furthermore, CXCL12–CXCR4 signaling pathways are also reported to be involved in MDSC trafficking in a breast tumor mouse model [67]. Moreover, CXCL12 produced by cancer associated fibroblasts can recruit MDSCs to the tumor microenvironment to exert tumor-promoting effects in mouse model of hepatic carcinoma [68] and estrogen receptor-negative breast cancer [69]. Therefore, CXCL12/CXCR4 interaction could be considered as an important driving force of MDSC recruitment into the tumor microenvironment. Thus, future immune-based strategies may be focused on combinations of different immune checkpoint inhibitors with substances targeting CXCL12/CXCR4 and reversal of local immunosuppression in the microenvironment, converting a ‘cold’ tumor into a ‘hot’ tumor [25,70].

Significantly, expression of CXCL12 and CXCR4 was observed in hypoxic regions but not in regions with proliferating cells [71,72,73]. Hypoxia induces expression of HIF-1α which activates transcription of VEGF and upregulates CXCL12, leading to recruitment of CXCR4^+^ bone marrow cells and neovasculogenesis [74,75]. A major component of these bone marrow-derived cells for neovasculogenesis is monocytes, which in the tumors differentiate into tumor-associated macrophages (TAMs). These TAMs promote tumor invasiveness, immune suppression and formation of new blood vessels [76,77]. 

**Figure 2:**
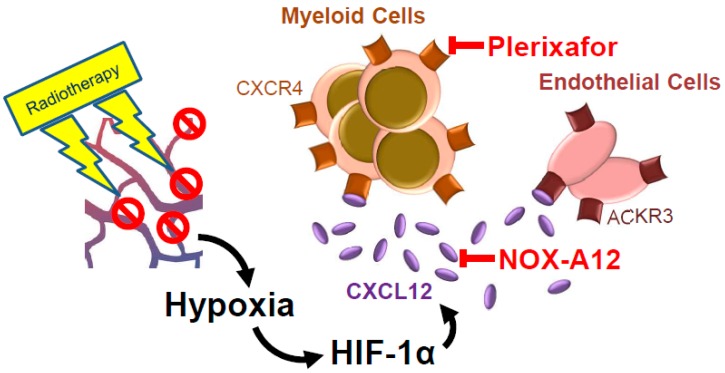
Radiotherapy increases hypoxia in glioblastoma by damaging the tumor vasculature. This leads to upregulation of CXCL12 via HIF-1α (hypoxia-induced factor-1α). Plerixafor (AMD3100) is representative of a number of different types of CXCR4 inhibitors. NOX-A12 binds to and inhibits CXCL12 directly.

Irradiation of GBM leads to loss of vasculature and increasing hypoxia [78,79] (Figure 2). Doses of 5–15 Gy stimulate vasculogenesis by upregulating CXCL12, in part via stimulation of HIF-1α [74,75]. Since recurrence of glioblastoma after radiotherapy relies on vasculogenesis, targeting CXCL12/CXCR4 was suggested as a novel therapeutic rationale in combination with radiotherapy in these tumors [79,80]. In a mouse GBM model, inhibition of CXCR4 with plerixafor (formerly known as AMD3100) prevented recruitment of bone marrow-derived cells (mainly CD11b^+^ cells), which together with irradiation (5x2Gy or 1x15Gy) prevented tumor recurrence [78], suggesting that these CD11b^+^ monocytes or their differentiated progeny, TAMs, supply signals for the formation of new vessels. This was corroborated by the ability of the CXCL12 antagonist NOX-A12, to control autochthonous brain tumors in rats. NOX-A12 is a so-called ‘Spiegelmer’, a class of synthetic L-ribonucleic acids that are designed to bind to specific targets conceptually similar to antibodies [81]. In this rat brain tumor model, which is extremely resistant to anticancer therapy, and which results in tumors with a genetic diversity and aggressiveness comparable to human brain tumors [82], NOX-A12 in combination with 20 Gy single dose irradiation significantly reduced tumor burden and prolonged rat survival even if the rats were irradiated when the tumors were already established [83]. In another model, NOX-A12 also enhanced the effect of the anti-VEGF antibody bevacizumab [84]. 

CXCL12 was also shown to recruit endothelial progenitor cells from the bone marrow to the tumor microenvironment where they form a new, functional vessel architecture that re-nourishes the tumor and propagates its regrowth [55]. Although some studies have reported that such ACKR3+ endothelial progenitor cells are supplied by the bone marrow, evidence from other studies point towards recruitment of circulating progenitor cells [79]. In fact, it could be shown experimentally that all components of the CXCL12/CXCR4/ACKR3 axis are relevant for recurrence of glioblastoma after radiotherapy, as inhibition of ACKR3 alone in combination with irradiation was also effective in preclinical models [85]. Furthermore, CXCL12-dependent vascularization turned out to be the most prominent bypass mechanism of VEGF-targeted therapy [86].

## 5. Function of the CXCL12-CXCR4 Axis in Brain and Glioblastoma

CXCL12 belongs to the evolutionary conserved CXC chemokine family expressed especially in the immune system, central nervous systems, and vasculature, of higher vertebrates [87]. CXCL12 has multiple functions that are essential during development and is perinatally lethal in knockout mice. Embryos show defective B-cell lymphopoiesis and myelopoiesis, abnormal neuron migration, and lacking or defective vasculature in kidneys, heart, and skin [88,89,90,91,92,93]. CXCL12 is expressed by different cell types including stromal cells in the bone marrow, glial and neuronal cells in the nervous system, and endothelial cells in various organs (reviewed in reference [87]). Six different splice variants in humans (CXCL12α to ϕ) are transcriptionally regulated in different cell types. The functions of their products are further regulated by posttranslational modification, including proteolytic truncation, citrullination of Arg residues, and nitration of Tyr residues (reviewed in reference [94]).

**Figure 3:**
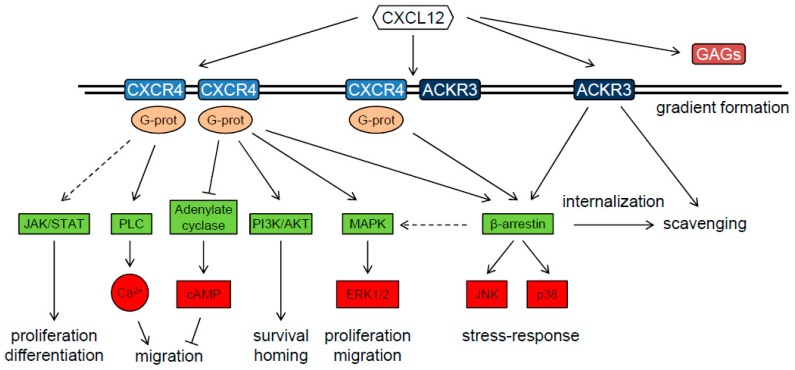
Schematic overview of the CXCL12-CXCR4-ACKR3 signaling cascade. Intermediate kinases have been omitted (for a detailed description, see reviews by Würth et al. [75] and Janssen et al. [94]). cAMP: cyclic adenosine-3’,5’-monophosphate; ERK1/2: extracellular signal-regulated kinase 1/2; GAG: glycosaminoglycans (heparin, heparan sulfate); G-prot: G-proteins; JAK/STAT: Janus kinase/signal transducers and activators of transcription; JNK: c-Jun N-terminal kinase; MAPK: mitogen-activated protein kinase; PI3K/AKT: phosphoinositide-3-kinase/Protein kinase B; PLC: phospholipase C.

CXCL12 is the only chemokine ligand binding to CXCR4, a heptahelical G-protein coupled receptor (GPCR) that is expressed on circulating leukocytes, hematopoietic progenitor and stem cells, as well as on endothelial, stromal and epithelial cells in various tissues [95]. Dissociation of G-protein subunits leads to intracellular signal transduction through MAP Kinase, PI3K/AKT, and phospholipase C pathways, inhibits cAMP signaling, and activates Ca^2+^ and K^+^ ion channels [75,87]. A further receptor for CXCL12 is atypical chemokine receptor (ACKR) 3 (formerly known as CXCR7). ACKRs are heptahelical membrane receptors that do not activate G-protein and are expressed mainly on non-leukocyte cell types, including vascular endothelial cells [96]. ACKR3 has a higher affinity for CXCL12 than CXCR4 and can act as a scavenger by internalization. However, it is also able to signal to MAP Kinase and p38 via β-arrestin [87,94] and has been reported to activate MAP Kinase via G protein in astrocytes and glioma cells [97]. In addition to membrane receptors, CXCL12 can bind to glycosaminoglycans (GAG) such as heparin sulfate found in the extracellular matrix and heparin secreted by mast cells, which is important for setting up chemokine gradients required for directed cell migration [94,98]. A schematic overview of the CXCL12/CXCR4/ACKR3 signaling cascade is shown in Figure 3. 

Homo-dimerization of CXCR4 and hetero-dimerization of CXCR4 with ACKR3 or other chemokine receptors determines the choice between multiple pathways leading to proliferation, migration, cell adhesion, or survival [75]. Thus, CXCL12 signaling via CXCR4 versus ACKR3 was shown to activate different transcriptional profiles, including two miRNAs [99]. 

An important function of the CXCL12/CXCR4/ACKR3 axis is regulation of migration and homing of stem and progenitor cells to their niches [75,100,101]. Thus, it regulates stem cells in the bone marrow as well as in the peripheral and central nervous system, and attracts lymphocytes and monocytes expressing the CXCR4 receptor [75]. In the brain, adult neural stem cells are found in vascular niches, and mesenchymal stem cells with potential for differentiating into astrocytes have been found in the perivascular niche [102]. Similarly, glioblastoma stem cells are found in perivascular niches [103] which have recently been reported to be hypoxic periarteriolar rather than pericapillary, and in which endothelial cells and smooth muscle cells provide signals required for maintenance [104]. CXCL12 is expressed by microvascular endothelial cells and stimulated proliferation of GBM progenitor cells but not differentiated tumor cells via CXCR4, whereas ACKR3 was not involved [105,106]. Furthermore, a CXCL12 gradient stimulates migration of GBM cells [107] by Ca^2+^-dependent activation of BK (Big-Potassium) K^+^ channels [108]. Notably, CXCL12-CXCR4 interactions target GBM stem cells to endothelial cells in the perivascular niche where they may be induced to differentiate into pericytes by transforming growth factor (TGF)-β1 [109]. 

Recent evidence supports the hypothesis human glioblastoma arises from neural stem cells in the subventricular zone (SVZ) [110] though, in a xenograft model, GBM cells have been found to migrate from the tumor to SVZ which was reported to constitute a CXCL12-dependent radioresistant niche [111]. However, the evidence for radioresistance is open to alternative interpretations and no direct evidence for an effect on cell survival was presented. Nevertheless, GBM stem cells are generally considered to show radioresistance mediated by intracellular cell signaling pathways and microenvironmental factors [112].

## 6. CXCL12 in Extracranial Tumors

The CXCL12/CXCR4/ACKR3 axis is not only implicated in GB, but also in extracranial tumors and is involved in tumor progression, angiogenesis, metastasis, and survival as well as contributing to immunosuppressive networks within the tumor microenvironment [113]. In hematological malignancies, the cross-talk among lymphoma, myeloma and leukemia cells and their microenvironments is mediated via CXCL12 and its receptors [114] and interference with CXCL12 signaling not only directly inhibits leukemic cell proliferation, but also mobilizes leukemic-lymphoma cells from their niches improving the efficacy of conventional treatments [115]. 

In solid malignancies, CXCL12 was shown to synergize with vascular endothelial growth factor (VEGF) to promote tumor angiogenesis [116]. CXCL12 also promotes tumor cell proliferation and survival [117] as well as invasion and metastasis via CXCR4 [118,119]. Interestingly, CXCR4+ tumor cells may have stem-like properties, a high metastatic potential, and show radiation resistance [120,121]. Some tumors also express ACKR3, which can promote adhesion, invasion, survival and growth [122,123]. 

Targeting the CXCL12/CXCR4 axis attenuated non-small cell lung cancer growth and augmented the effects of chemotherapy and radiotherapy [124]. In pancreatic cancer, inhibition of the CXCL12/CXCR4 axis arrested cell growth and abrogated gemcitabine resistance [125] as well as reduced tumor growth in animal models by blocking CXCR4-dependent mast cell migration [126]. CXCL12/CXCR4 axis blockade in combination with sorafenib treatment was reported to inhibit hepatocellular cancer growth [127]. Blocking the CXCR4/CXCL12 axis also inhibited tumor growth and metastasis formation in breast cancer models [128,129]. In models of prostate cancer, inhibition of CXCR4-dependent vascularization delayed tumor growth [130]. In addition, inhibition of CXCL12/CXCR4 signaling by antibodies, peptide analogues, or small molecules has been found to reduce metastatic burden in various orthotopic and metastatic mouse xenograft models [131,132,133]. 

Recently, it was reported that T cells may be excluded from cancer cell nests by high CXCL12 levels. Consequently, blocking the CXCL12/CXCR4 axis improved immuno-oncological approaches such as checkpoint inhibition in models of pancreatic cancer [134] and hepatocellular cancer [135]. Clinically, patients with cervical cancer treated with standard-of-care radio-chemotherapy and the CXCR4 inhibitor plerixafor showed improved primary tumor response and reduced metastases [136]. 

## 7. Current CXCL12/CXCR4/ACKR3 Inhibitors 

A number of antagonists and inhibitors targeting the CXCL12/CXCR4/ACKR3 axis are being developed in cancer indications, mostly in non-brain tumor related cancers and not in combination with radiotherapy (Table 1). Whereas three agents—plerixafor, NOX-A12 and USL311—have been or are reported to be tested in glioblastoma, only the first two are currently explored in combination with radiotherapy, thus targeting the angiogenic-vasculogenic ‘switch’ induced by radiotherapy. Plerixafor is a bicyclam molecule that antagonizes the CXCR4 receptor. It rapidly and reversibly mobilizes hematopoietic stem cells into the peripheral circulation and was first approved in combination with granulocyte-colony stimulating factor (G-CSF) to mobilize hematopoietic stem cells to the peripheral blood for collection and subsequent autologous transplantation in patients with hematological malignancies. 

This approach is currently being tested in a Phase 1/2 study in adult patients with newly diagnosed glioblastoma assessing the impact of CXCR4 blockade with plerixafor combined with radiotherapy and temozolomide (NCT01977677). Interim data presented at ASCO 2018, with 29 patients enrolled, validate the concept of targeting the axis. This study showed that inhibition of the CXCL12/CXCR4 axis (i) is safe in brain tumor patients, (ii) leads to improved local control, (iii) may improve overall survival with an estimated median OS of 20.7 months, and (iv) shifts the pattern of recurrence towards an out-of-field pattern (58.8% out-of-field recurrences vs. 10% in a control group) [137]. This study therefore validates preclinical data by demonstrating that inhibition of the CXCL12/CXCR4 pathway improves local control of GBM following radiotherapy [78,83]. Another study with plerixafor was performed in recurrent high-grade glioma (HGG) patients in combination with VEGF inhibition but without concomitant radiotherapy. This approach tested whether blocking CXCR4 could break the resistance of the tumor to anti-angiogenic therapy. However, while confirming good safety and tolerability of the combination in these patients, the effect on the clinical outcome of these particularly hard-to-treat patients was limited [138].

Targeting the ligand, CXCL12, rather than its receptor, CXCR4, NOX-A12 is another inhibitor of the axis that is to be tested in brain tumors in combination with radiotherapy. The compound was previously tested in two phase I studies in healthy volunteers and two phase IIa studies, one in multiple myeloma patients [139], and one in patients with chronic lymphocytic leukemia. Another phase I/II study in patients with pancreatic and colorectal cancer in combination with PD-1 checkpoint inhibition is ongoing (Keynote-559; NCT03168139). The translational relevance of the preclinical data generated with NOX-A12 described earlier [83] is supported by clinical correlative studies showing that elevated expression of CXCL12 and its receptors, CXCR4 and ACKR3, is associated with higher tumor grade and invasion as well as decreased apoptosis in glioblastoma [140,141]. 

## 8. Conclusions

The CXCL12/CXCR4 axis is a key regulator of the post-radiotherapy microenvironment in glioblastoma. Blocking of CXCL12 or CXCR4 could prevent the influx of tumor associated macrophages and myeloid suppressor cells which mediate re-vascularization and immunosuppression respectively. Initial results from an early clinical trial warrant further investigation of this therapeutic approach in glioblastoma. 

## Figures and Tables

**Table 1:** Antagonists of the CXCL12/CXCR4/ACKR3 axis currently in clinical development for cancer.

Compound	Company	Type of Molecule	Target	Indications and Study Pase
**Mozobil^®^** **(plerixafor, AMD3100)**	Sanofi-Genzyme	Small molecule	CXCR4	Stem cell mobilization: approved Multiple myeloma: Phase 1/2 Glioblastoma: Phase 1/2 Advanced solid tumors (pancreas, ovarian, colorectal): Phase 1 AML/ALL: Phase 1
**BL-8040**	BioLineRx	Peptide	CXCR4	Stem cell mobilization: Phase 3 AML*: Phase 2 Metastatic pancreatic cancer*: Phase 2 T-ALL: Phase 2 Advanced gastric cancer*: Phase 1/2 Metastatic non-small cell lung cancer*: Phase 1/2
**NOX-A12** **Olaptesed pegol**	NOXXON Pharma	L-RNA, PEGylated	CXCL12	Metastatic colorectal and pancreatic cancer: Phase 1/2* Glioblastoma: Phase 1/2
**Burixafor** **TG-0054**	TaiGen	Small molecule	CXCR4	Metastatic prostate cancer: Phase 1
**LY-2510924**	Eli Lilly	Peptide	CXCR4	AML: Phase 1 *Discontinued:* *Metastatic clear cell renal cell carcinoma combined with sunitinib (Phase 2)* *Extensive-disease small-cell lung cancer combined with carboplatin/etoposide (Phase 2)*
**Balixafortide** **POL6326**	Polyphor	Peptide	CXCR4	Metastatic breast cancer: Phase 1
**Ulocuplumab** **BMS 936564**	BMS	Monoclonal antibody	CXCR4	Waldenström’s macroglobulinemia: Phase 1/2 AML: Phase 1/2 *Discontinued:* *Advanced solid tumors (pancreatic and lung cancer patients) combined with nivolumab (Phase 1/2)*
**X4P-001**	X4 Pharmaceuticals	Small molecule	CXCR4	Metastatic clear cell renal cell carcinoma*, **: Phase 1/2 Resectable melanoma*: Phase 1
**USL311**	Proximagen	Small molecule	CXCR4	Recurrent glioblastoma: Phase 2

*plus PD-1/PD-L1 inhibitor; **plus axitinib. ALL—acute lymphoblastic leukemia; AML—acute myeloid leukemia; CLL—chronic lymphocytic leukemia; MM—multiple myeloma.
